# O Impacto da COVID-19 no Diagnóstico de Doenças Cardíacas na América Latina Uma Subanálise do INCAPS COVID

**DOI:** 10.36660/abc.20210388

**Published:** 2022-01-11

**Authors:** Rodrigo Julio Cerci, João Vicente Vitola, Diana Paez, Alejandro Zuluaga, Marcio Sommer Bittencourt, Lilia M. Sierra-Galan, Patricia Carrascosa, Roxana Campisi, Claudia Gutierrez-Villamil, Amalia Peix, Duane Chambers, Mayra Sánches Velez, Carla M. G. Alvarado, Ana C. F. Ventura, Alejandro Maldonado, Alfredo P. Castanos, Teresa C. Diaz, Yariela Herrera, Manuel C. Vasquez, Ana A. Arrieta, Fernando Mut, Cole Hirschfeld, Eli Malkovskiy, Benjamin Goebel, Yosef Cohen, Michael Randazzo, Leslee J. Shaw, Michelle C. Williams, Todd C. Villines, Nathan Better, Sharmila Dorbala, Paolo Raggi, Thomas N. B. Pascual, Yaroslav Pynda, Maurizio Dondi, Andrew J. Einstein

**Affiliations:** 1 Quanta Diagnóstico por Imagem Curitiba PR Brasil Quanta Diagnóstico por Imagem - Cardiovascular CT, Curitiba , PR - Brasil; 2 International Atomic Energy Agency Division of Human Health Vienna Áustria International Atomic Energy Agency - Division of Human Health , Vienna - Áustria; Cedimed Quirosalud Universidad Pontificia Bolivariana Colômbia Cedimed Quirosalud , Universidad Pontificia Bolivariana y Colômbia; 3 Universidad CES Medelin Colômbia Universidad CES , Medelin - Colômbia; 4 Diagnósticos da América SA Barueri SP Brasil Diagnósticos da América SA – DASA, Barueri , SP - Brasil; 5 American British Cowdray Medical Center IAP Mexico City México American British Cowdray Medical Center IAP , Mexico City , Mexico City - México; 6 Diagnostico Maipu Buenos Aires Argentina Diagnostico Maipu , Buenos Aires - Argentina; 7 Instituto Argentino de Diagnostico y Tratamiento S.A Buenos Aires Argentina Instituto Argentino de Diagnostico y Tratamiento S.A , Buenos Aires - Argentina; 8 Fundación Cardioinfantil Instituto de Cardiologia Bogota Colômbia Fundación Cardioinfantil - Instituto de Cardiologia , Bogota - Colômbia; 9 Institute of Cardiology and Cardiovascular Surgery Department of Nuclear Medicine La Habana Cuba Institute of Cardiology and Cardiovascular Surgery - Department of Nuclear Medicine , La Habana - Cuba; 10 Radiology West Montego Bay Jamaica Radiology West , Montego Bay - Jamaica; 11 Carlos Andrade Marin Specialty Hospital Quito Equador Carlos Andrade Marin Specialty Hospital , Quito - Equador; 12 Tecnodiagnosis Guatemala Guatemala Tecnodiagnosis , Guatemala - Guatemala; 13 Radiology Clinic Brito Mejia Pena San Salvador El Salvador Radiology Clinic Brito Mejia Pena , San Salvador - El Salvador; 14 Servicio de Medicina Nuclear Tegucigalpa Honduras Servicio de Medicina Nuclear , Tegucigalpa - Honduras; 15 Centro Diagnostico Especializado Santo Domingo República Dominicana Centro Diagnostico Especializado , Santo Domingo - República Dominicana; 16 Hospital Salud Integral Panama City Panamá Hospital Salud Integral , Panama City - Panamá; 17 Hospital Santo Tomas Nuclear Medicine Section Panamá Hospital Santo Tomas - Nuclear Medicine Section , Panama - Panamá; 18 Hospital Central Instituto de Prevision Social Asuncion Paraguai Hospital Central , Instituto de Prevision Social , Asuncion - Paraguai; 19 Hospital Dr. Rafael Angel Calderon Guardia San Jose Costa Rica Hospital Dr. Rafael Angel Calderon Guardia , San Jose - Costa Rica; 20 Italian Hospital Nuclear Medicine Service Montevideo Uruguai Italian Hospital - Nuclear Medicine Service , Montevideo - Uruguai; 21 Columbia University Irving Medical Center New York-Presbyterian Hospital Department of Medicine New York Estados Unidos da América Columbia University Irving Medical Center and New York-Presbyterian Hospital - Department of Medicine , New York - Estados Unidos da América; 22 Paul and Gloria Milstein Division of Cardiology New York Estados Unidos da América Seymour, Paul and Gloria Milstein Division of Cardiology , New York - Estados Unidos da América; 23 Weill Cornell Medical College New York-Presbyterian Hospital Estados Unidos da América Weill Cornell Medical College and New York-Presbyterian Hospital ,New York - Estados Unidos da América; 24 Technion Israel Institute of Technology Haifa Israel Technion Israel Institute of Technology , Haifa - Israel; 25 BHF Centre for Cardiovascular Science University of Edinburgh Edinburg Reino Unido da Grã-Bretanha BHF Centre for Cardiovascular Science , University of Edinburgh , Edinburg - Reino Unido da Grã-Bretanha; 26 University of Virginia Charlottesville Virginia Estados Unidos da América University of Virginia , Charlottesville , Virginia - Estados Unidos da América; 27 Royal Melbourne Hospital University of Melbourne Melbourne Austrália Royal Melbourne Hospital and University of Melbourne , Melbourne - Austrália; 28 Brigham and Women’s Hospital Boston Massachusetts Estados Unidos da América Brigham and Women’s Hospital , Boston , Massachusetts - Estados Unidos da América; 29 University of Alberta Department of Medicine Division of Cardiology Edmonton Alberta Canadá University of Alberta - Department of Medicine and Division of Cardiology , Edmonton , Alberta - Canadá; 30 Philipines Nuclear Research Institute Manila Filipinas Philipines Nuclear Research Institute , Manila - Filipinas; 31 International Atomic Energy Agency Division of Human Health Vienna Áustria International Atomic Energy Agency - Division of Human Health , Vienna - Áustria

**Keywords:** Teste Cardíaco, Coronavírus, COVID-19, Doença Cardiovascular, Saúde global

## Abstract

**Fundamento:**

A pandemia de COVID-19 interferiu na prestação de atendimento a doenças cardiovasculares na América Latina. No entanto, o efeito da pandemia nos volumes de procedimentos cardíacos diagnósticos ainda não foi quantificado.

**Objetivo:**

Avaliar (1) o impacto de COVID-19 nos volumes de diagnóstico cardíaco na América Latina e (2) determinar sua relação com a incidência de casos de COVID-19 e as medidas de distanciamento social.

**Métodos:**

A International Atomic Energy Agency realizou uma pesquisa mundial avaliando mudanças nos volumes diagnósticos cardíacos decorrentes da COVID-19. Foram obtidos os volumes diagnósticos cardíacos dos locais participantes para março e abril de 2020 e comparados com março de 2019. Foram coletados dados de distanciamento social a partir dos Relatórios de mobilidade da comunidade de Google e a incidência de COVID-19 por país a partir de Our World in Data.

**Resultados:**

Foram realizadas pesquisas em 194 centros que realizam procedimentos diagnósticos cardíacos, em 19 países da América Latina. Em comparação com o mês de março de 2019, os volumes dos procedimentos diagnósticos cardíacos diminuíram 36% em março de 2020 e 82% em abril de 2020.As maiores reduções ocorreram em relação aos testes de estresse ecocardiográfico (91%), testes ergométricos de esteira (88%) e escore de cálcio por tomografia computadorizada (87%), com pequenas variações entre as sub-regiões da América Latina. As mudanças em padrões de distanciamento social (p < 0,001) estavam mais fortemente associadas com a redução do volume do que a incidência de COVID-19 (p = 0,003).

**Conclusões:**

A COVID-19 foi associada a uma redução significativa de procedimentos diagnósticos cardíacos na América Latina, a qual foi mais relacionada ao distanciamento social do que ao aumento da incidência da COVID-19. São necessários melhor equilíbrio e timing de medidas de distanciamento social e planejamento para manter o acesso ao atendimento médico durante um surto pandêmico, especialmente em regiões com alta mortalidade cardiovascular.

## Resumo curto

A pandemia de COVID-19 foi associada a uma redução significativa de procedimentos diagnósticos cardíacos na América Latina em abril de 2020, a qual foi mais relacionada às medidas distanciamento social do que ao aumento da incidência da COVID-19.

## Pontos principais

- Em comparação com o mês de março de 2019, os volumes dos procedimentos diagnósticos cardíacos diminuíram 36% em março de 2020 e 82% em abril de 2020.
- As mudanças em padrões de distanciamento social (p < 0,001) estavam mais fortemente associadas com a redução volume do que a incidência de COVID-19 (p = 0,003).
- São necessários melhor equilíbrio e timing de medidas de distanciamento social e planejamento para manter o acesso ao atendimento médico durante um surto pandêmico, especialmente em regiões com alta mortalidade cardiovascular.

## Introdução

As doenças cardiovasculares (DCV) continuam sendo a causa principal de mortalidade em todo o mundo, inclusive na América Latina. ^1 , 2^ Embora as taxas de mortalidade tenham diminuído progressivamente nas últimas quatro décadas na maioria dos países de alta renda, o mesmo fenômeno não foi observado nos países de baixa e média renda, muitos dos quais se encontram na América Latina. ^3^

Uma abordagem abrangente para lidar com as DCV e reduzir a mortalidade associada envolve prevenção adequada, incluindo controle de fatores de risco, uso apropriado de testes para diagnosticar e orientar o tratamento e o estabelecimento de terapias apropriadas. A Organização Mundial da Saúde recentemente chamou a atenção para a interrupção mundial da assistência à saúde causada pela pandemia de COVID-19, que infelizmente impõe uma carga adicional ao atendimento de pacientes com DCV em regiões como a América Latina que têm sido gravemente afetadas pela COVID-19. ^4^

A International Atomic Energy Agency (IAEA) Division of Human Health visa apoiar os estados membros no combate às DCV, câncer, desnutrição e outras doenças por meio de prevenção, testes diagnósticos e tratamento adequados. Nesse sentido, a IAEA coordenou um levantamento mundial de centros de imagem cardiovascular (IAEA *Noninvasive Cardiology Protocols Study of COVID-19, INCAPS COVID survey* ), com a finalidade de avaliar o impacto da pandemia na avaliação diagnóstica das DCV.

Os objetivos deste estudo foram: (1) avaliar o impacto da COVID-19 nos volumes de procedimentos diagnósticos cardíacos na América Latina e (2) determinar sua relação com a incidência de casos de COVID-19, apresentação temporal e intervenções de distanciamento social. Compreender a relação entre as fases da pandemia, as medidas de distanciamento social e o fornecimento de diagnóstico de DCV na América Latina é fundamental para melhor preparar para situações semelhantes no futuro.

## Métodos

### Desenho do estudo

Os dados deste estudo foram coletados como parte da pesquisa da IAEA sobre o impacto da COVID-19 em exames de imagem cardíaca (INCAPS COVID) e correlacionados com as métricas de distanciamento social que estão publicamente disponíveis nos Relatórios de mobilidade da comunidade de Google e a incidência mensal de COVID-19 a partir do banco de dados Our World in Data na América Latina. ^5 - 7^ A pesquisa INCAPS COVID incluiu perguntas sobre a unidade de saúde, os profissionais de saúde, os equipamentos de proteção individual, os planos estratégicos para reabertura e as alterações nos volumes de procedimentos em relação a uma série de procedimentos diagnósticos cardiovasculares (Apêndice).

### Coleta de dados

Com base na metodologia padronizada da IAEA, foi criado um sistema eletrônico de entrada de dados, empregando uma plataforma segura de software, o International Research Integration System (IRIS, https://iris.iaea.org). No estudo INCAPS COVID, não foram coletados dados confidenciais ou específicos dos pacientes e a participação dos locais de estudo foi voluntária; portanto, não foi considerada necessária a avaliação por comitê de ética externo.

Os participantes foram solicitados a fornecer estimativas dos volumes de procedimentos diagnósticos cardíacos para março de 2019, março de 2020 e abril de 2020, incluindo os seguintes: ecocardiografia transtorácica e transesofágica, ressonância magnética cardíaca (RMC), teste de estresse (teste ergométrico de esteira, teste de estresse ecocardiográfico, tomografia computadorizada por emissão de fóton único [SPECT], tomografia por emissão de pósitrons [PET] e RMC), estudos de infecção por PET, escore de cálcio por tomografia computadorizada, angiotomografia de artérias coronárias e angiografia coronária invasiva. Para fins de análise, dividimos a América Latina nas seguintes sub-regiões: América do Sul (Argentina, Bolívia, Brasil, Chile, Colômbia, Equador, Paraguai, Peru e Uruguai); América Central e México (Costa Rica, Guatemala, Honduras, México, Nicarágua, Panamá e El Salvador); e Caribe (Cuba, Jamaica e República Dominicana).

### Casos de COVID-19 por país

Os números de casos de COVID-19 para cada país da América Latina foram baixados do site de acesso aberto Our World in Data (https://ourworldindata.org/coronavirus-source-data), que coleta dados de diferentes fontes oficiais em todo o mundo. ^5^ Our World in Data é um trabalho colaborativo entre pesquisadores da Universidade de Oxford baseado no Oxford Martin Programme on Global Development, que são os editores científicos do conteúdo do site, e a organização sem fins lucrativos Global Change Data Lab, que publica e mantém o site e as ferramentas de dados. Os dados coletados abrangem o período de fevereiro de 2020 a julho de 2020 para melhor refletir a evolução da pandemia na América Latina. Foi utilizado para análise o número de casos novos a cada mês por milhão de habitantes.

### Dados de mobilidade por país

Os dados de mobilidade foram baixados dos Relatórios de mobilidade da comunidade de Google (https://www.google.com/covid19/mobility/) que agregam as tendências de mobilidade em 6 categorias diferentes: varejo e lazer, mercados e farmácias, parques, estações de transporte público, locais de trabalho e residencial. A linha de base foi o valor mediano, para o dia da semana correspondente, durante o período de 5 semanas entre o dia 3 de janeiro e o dia 6 de fevereiro de 2020. ^6^ Google calcula essas informações com base nos dados de usuários que optaram pelo histórico de localização por meio de suas contas de Google; portanto, os dados representam uma amostra de todos os usuários. Utilizou-se a variação do tempo passado em casa (residencial) por mês como uma variável do “índice de imobilidade”, de fevereiro de 2020 a julho de 2020, que reflete mudanças nos padrões de distanciamento social durante esse período. Os dados de mobilidade de Cuba não estavam disponíveis e, portanto, não foram utilizados nessas análises.

### Análise estatística

As respostas às perguntas da pesquisa são apresentadas como números e porcentagens. O total de procedimentos por centro é apresentado como mediana e intervalo interquartil. A mudança percentual no volume de procedimentos foi comparada entre março de 2019 e março ou abril de 2020 utilizando o teste não paramétrico de Kruskal-Wallis com valores de p assintóticos e bilaterais. Para avaliar a associação das mudanças nos volumes de procedimentos cardíacos com as mudanças na mobilidade e a incidência da COVID-19, construímos uma equação de estimativa generalizada utilizando os países como unidades individuais e os números mensais de exames como o desfecho com o mês como variável de tempo. Foi realizada a análise estatística com Stata (versão 15.1, Stata Corporation, LLC, College Station, Texas), Microsoft Excel (2016) e foram construídos mapas coropléticos em R (versão 4.0.1, R Development Core Team, Viena, Áustria) utilizando os pacotes tmap e rnaturalearth.

## Resultados

### Centros e redução de procedimentos

Foram obtidos dados de 194 centros de internação e ambulatórios em 19 países da América Latina. Os maiores países regionais Brasil, Argentina e México também foram os que contribuíram com dados do maior número de centros: 70, 54 e 23 centros, respectivamente. As características de todos os centros estão resumidas na Tabela 1 . Em total, foram realizados 198.597 procedimentos diagnósticos cardíacos nos centros participantes durante os três meses considerados.

**Tabela 1:** – Características dos centros da América Latina

	América do Sul	América Central e México	Caribe
**Países**	**9**	**7**	**3**
**Número de centros**	**155**	**31**	**8**
**Instituto de ensino (n, %)**	**69 (44,5)**	**21 (67,7)**	**4 (50)**
**Leitos hospitalares (mediana, IIQ)**	**202,5 (120 - 400)**	**167 (100 - 300)**	**168 (80 – 412,5)**
**Tipo de instituto (n, %)**			
Hospital, apenas internamento	3 (1,9)	5 (16,1)	0 (0)
Hospital, apenas ambulatório	3 (1,9)	0(0)	0 (0)
Hospital, internação e ambulatório	91 (58,7)	19 (61,3)	4 (50)
Centro de imagem ambulatorial	45 (29,0)	2 (6,4)	2 (25)
Consultório médico ambulatorial	13 (8,4)	5 (16,1)	2 (25)
**Procedimentos totais por centro (mediana, IIQ)**			
Março 2019	157 (67 - 502)	91 (38 - 430)	173 (53,5 - 559,5)
Março 2020	89 (31 - 253)	35 (19 - 143)	147,5 (24,5 - 343,5)
Abril 2020	32 (7 - 97)	20 (1 - 53)	36 (2,5 - 117)
**% Redução de março de 2019 a abril de 2020**			
Ecocardiografia transtorácica	80,1	54,0	87,0
Ecocardiografia transesofágica	81,6	88,0	89,6
RMC	77,2	80,9	100
Escore de cálcio por TC	81,7	96,1	99,5
TC coronária	73,0	84,8	85,9
Angiografia coronária invasiva	63,8	77,7	70,8
Teste ergométrico de esteira	88,4	84,9	95,6
Teste de estresse ecocardiográfico	91,1	94,5	76,9
SPECT	84,0	81,0	97,8
PET	62,0	90,9	NA
RMC de estresse	76,3	89,2	NA

*IIQ: intervalo interquartil; PET: tomografia por emissão de pósitrons; RMC: ressonância magnética cardíaca; SPECT: tomografia computadorizada por emissão de fóton único; TC: tomografia computadorizada.*

**Figura 1:**
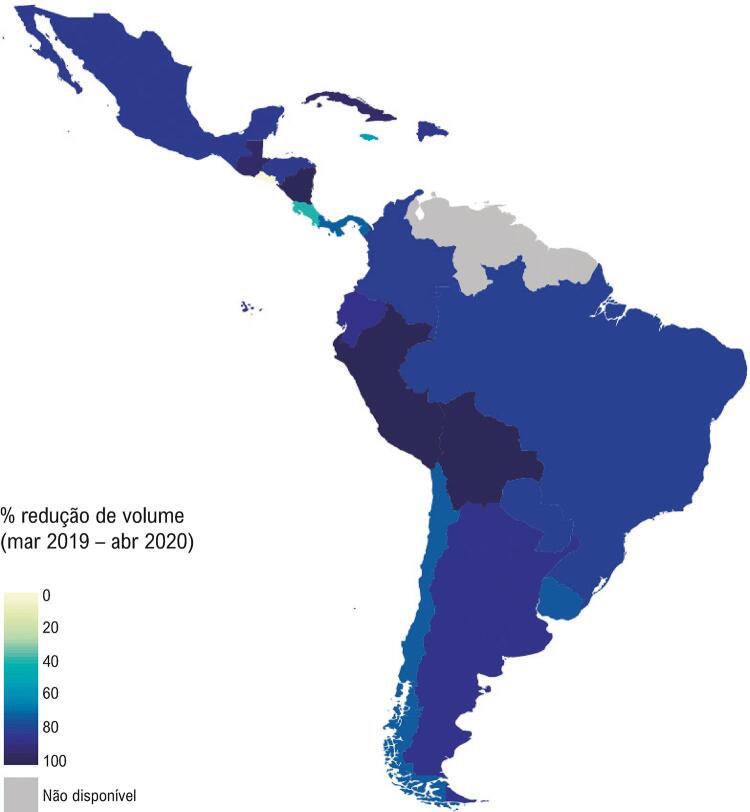
– Mapa com código de cores da América Latina mostrando a redução dos volumes totais de procedimentos diagnósticos cardíacos por país de março de 2019 a abril de 2020, no começo da pandemia de COVID-19.

**Figura 2:**
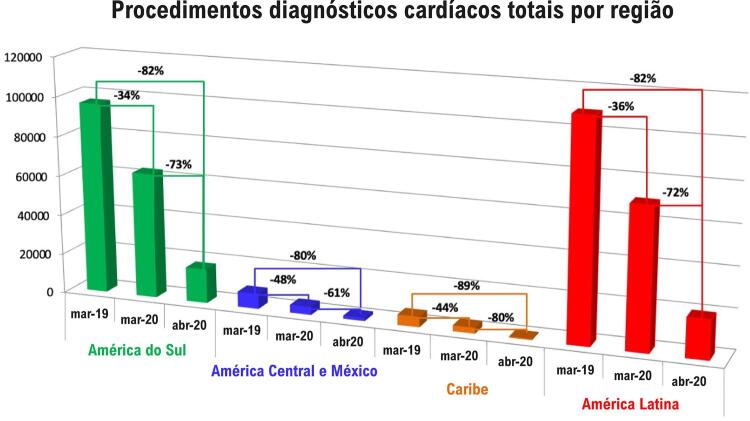
– Redução dos volumes totais de procedimentos diagnósticos cardíacos em sub-regiões da América Latina: América do Sul; América Central e México; e Caribe, em março de 2019, março de 2020 e abril de 2020, no começo da pandemia de COVID-19.

Na América Latina, em comparação com o mês de março de 2019, os volumes dos procedimentos diagnósticos cardíacos diminuíram 36% em março de 2020 e 82% em abril de 2020 ( Figura 1 – Mapa). Houve alguma variação entre as regiões da América Latina e em relação ao tipo de procedimento cardíaco, com as maiores quedas no mês de abril de 2020, em relação aos testes de estresse ecocardiográfico (91%), testes ergométricos de esteira (88%) e escore de cálcio por tomografia computadorizada (87%) ( Tabela 1 ). Foram relatadas as menores reduções para angiografia coronária invasiva (67%) e PET cardíaca (65%). Os volumes de procedimento também diminuíram acentuadamente de março de 2020 a abril de 2020. Essas diminuições foram significativas (p < 0,001) em combinação ( Figura 2 ) e para cada procedimento. Modelos lineares generalizados separados para as regiões da América Latina e em geral encontraram quedas significativas no volume de procedimentos (p < 0,001), usando modelos de regressão ponderados pelo volume de procedimentos de 2019. Nas 194 instalações de nosso estudo, estima-se que 129.030 procedimentos diagnósticos cardíacos, que teriam sido realizados com base nas taxas de procedimentos de março de 2019, não foram realizados durante esses dois meses da pandemia.

### Dados de mobilidade, COVID-19 incidência e redução no volume de procedimentos

**Figura 3:**
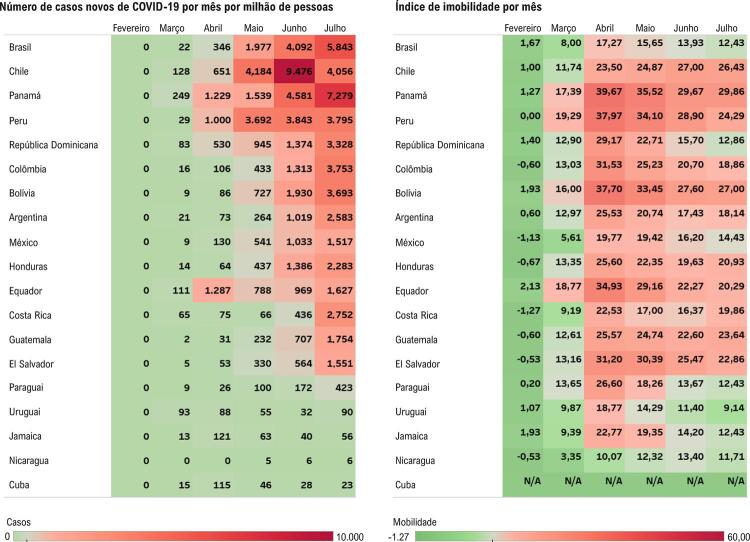
– Painel direito: Número de casos novos de COVID-19 por mês por milhão de pessoas em 19 países da América Latina. Painel esquerdo: Mudança no tempo passado em casa (“índice de imobilidade”) por mês, utilizando o período de 5 semanas do dia 3 de janeiro ao dia 6 de fevereiro de 2020 como linha de base em 18 países da América Latina (com exceção de Cuba). Nota-se que a maior imobilidade ocorreu em abril de 2020 na maioria dos países, em concordância com a queda abrupta dos procedimentos diagnósticos cardíacos. Por outro lado, o número de casos novos de COVID-19 por milhão de pessoas ainda estava aumentando progressivamente de março a julho de 2020 na maioria dos países.

O maior aumento de tempo em casa (índice de imobilidade) ocorreu durante o mês de abril de 2020 na maioria dos países. Houve aumento médio de 26,7% em abril que diminuiu para 18,8% em julho, em comparação com a linha de base. As exceções foram Nicarágua e Chile, onde a imobilidade aumentou até junho ( Figura 3 ).

No entanto, a pandemia de COVID-19 ainda estava em seus estágios iniciais na maioria dos países em abril de 2020, com um total de 199.277 casos até o final do mês. Na maioria dos países, os novos casos mensais de COVID-19 por milhão de habitantes continuaram aumentando e ainda não haviam atingido o pico em julho de 2020 ( Figura 3 ). As exceções foram Cuba e Jamaica, que atingiram o pico em abril. Até o final de julho, o número de casos aumentou 23,5 vezes, totalizando 4.681.377 casos confirmados de COVID-19. ^5^

Tanto a redução da mobilidade quanto o aumento da incidência de COVID-19 estiveram associados à redução dos procedimentos diagnósticos cardíacos nos modelos generalizados (p < 0,001). Ao ajustar um modelo multivariável, a mobilidade (p < 0,001) foi mais fortemente associada à redução de volume do que a incidência de COVID-19 (p = 0,003).

## Discussão

Os resultados mostram uma redução significativa no número de procedimentos diagnósticos cardíacos realizados na América Latina durante a pandemia de COVID-19. A maior redução no número de procedimentos diagnósticos ocorreu no mês de menor mobilidade (abril), que por sua vez coincidiu com os períodos mais rígidos de quarentena de cada país.

Na maioria dos países da América Latina, o isolamento social foi introduzido em março, mesmo sem um número significativo de casos COVID-19. Em março e abril, houve uma queda maior no número de procedimentos diagnósticos cardíacos realizados na América Latina, em comparação com a Europa Ocidental (queda de 46% em março e 69% em abril) e os Estados Unidos e Canadá (queda de 39% em março e 68% em abril), apesar do fato de que estas regiões estavam experimentando o primeiro pico da pandemia, enquanto a América Latina estava apenas nos estágios iniciais. ^7^ Até o final de março, haviam sido notificados menos de 250 casos em 9 países da região e, desde então, o número de casos continuou aumentando. Até o final de agosto de 2020, o SARS-CoV-2 havia comprometido todos os países da América Latina, com 7,15 milhões de pessoas afetadas. ^5^ O número real de casos pode ser ainda maior, já que o número de exames por milhão de pessoas continua baixo. ^8 , 9^

Além da mortalidade direta causada pela COVID-19, houve preocupações crescentes em relação às consequências da pandemia de COVID-19 nos sistemas de saúde. ^10 - 12^ O medo do contágio nos hospitais e centros de saúde pode ter levado à relutância dos pacientes em se submeter a procedimentos de diagnóstico cardíaco. Além disso, as intervenções e consultas eletivas tiveram que ser adiadas para priorizar as questões relacionadas à COVID-19 e evitar a exposição dos pacientes a um risco desnecessário de infecção em ambientes hospitalares ou ambulatoriais. ^13^

A América Latina frequentemente enfrenta problemas de saúde que afetam principalmente os pobres, além de sistemas de saúde que já estão frágeis. ^14 , 15^ Nessas condições, o distanciamento social retarda o pico da pandemia para permitir que os países com recursos de saúde limitados preparem-se para o diagnóstico e tratamento de pacientes em estado crítico. ^16 , 17^ Apesar disso, de acordo com Walker e colegas, os países da América Latina que tiveram o primeiro pico da pandemia (Equador, México, Brasil, Chile, Bolívia, Panamá, Peru) ou com curvas de saúde não mitigadas/limitadas (Peru, Chile, México, Equador) registraram uma taxa de mortalidade mais alta por milhão de habitantes. ^18^ Isso poderia ser explicado pela incapacidade desses países de se prepararem para o pico da pandemia, com seus sistemas de saúde vulneráveis, resultando assim em altos índices de mortalidade. Eventos semelhantes ocorreram em países europeus atingidos pela primeira onda da pandemia de COVID-19, como a Itália e a Espanha.

No nível global, aproximadamente 70% das mortes por DCV ocorrem em países de baixa e média renda. ^3 , 19^ O grande intervalo de tempo entre o começo das medidas de distanciamento social e o primeiro pico da pandemia nos países da América Latina limitou o acesso dos pacientes aos procedimentos diagnósticos cardíacos, retardando ainda mais o diagnóstico e o tratamento oportuno das DCV. Na população da América Latina, essa cascata de eventos pode aumentar a morbimortalidade cardiovascular, conforme já tem sido relatado no Brasil. ^20^ A presente pesquisa não foi projetada para coletar informações sobre desfechos, mas o efeito negativo dos atrasos diagnósticos provavelmente será corroborado em estudos futuros para tais fins. Foi alcançada uma conclusão semelhante pela Sociedade Latino-Americana de Cardiologia Intervencionista que realizou um estudo sobre a prática da Cardiologia Intervencionista durante a pandemia de COVID-19, com foco no infarto do miocárdio. ^21^ Relataram uma redução de 51,2% no atendimento para infarto do miocárdio com elevação do segmento ST, com risco de aumento da morbimortalidade subsequente.

A infecção por COVID-19 pode estar associada a eventos cardiovasculares ou mimetizar doença cardíaca. ^22 - 27^ Portanto, é essencial, durante a pandemia de COVID-19, manter a disponibilidade de todas as modalidades de diagnóstico cardíaco, em pacientes tanto positivos quanto negativos para COVID-19.

Existem várias lições que aprendemos para o futuro. Destacamos cinco: 1) Durante uma pandemia, deve ser mantido o acesso aos procedimentos diagnósticos cardíacos, tanto quanto possível, para toda a população, independentemente do tipo de restrição de mobilidade estabelecida em cada país, seguindo rigorosamente os devidos cuidados sanitários. 2) Campanhas educativas devem ser estabelecidas na mídia e nas redes sociais para explicar à comunidade a importância de buscar ajuda rapidamente diante dos sinais de alerta de doenças cardíacas, ao mesmo tempo em que se implementam medidas para prevenir a disseminação da COVID-19. 3) Áreas não COVID (“azul”) para o atendimento de patologias não COVID e áreas para COVID (“vermelho”) para pacientes infectados devem ser estabelecidas nos serviços de saúde. 4) É necessário garantir o acesso mundial aos suprimentos de saúde, de equipamentos de proteção individual a radiotraçadores. ^28^ 5) Os governos devem garantir serviços de saúde não apenas aos pacientes com COVID, mas também aos pacientes sem COVID mas com DCV.

### Limitações

A pesquisa INCAPS COVID-19 avaliou dados dos meses de março e abril de 2020, quando a pandemia ainda estava em fase inicial na maioria dos países da América Latina. Porém, de acordo com a evolução dos *lockdowns* , as datas de reabertura econômica e os dados de mobilidade de cada país, abril de 2020 foi o mês de menor atividade nesses países. A pesquisa foi realizada em um número limitado de hospitais e centros diagnósticos de cada país, com participação variável, o que poderia colocar em questão a representabilidade dos resultados. Apesar isso, a redução universal do número de procedimentos cardíacos não invasivos realizados em toda a América Latina sugere que a nossa amostra é representativa. Finalmente, dados de pesquisa de longo prazo para acompanhar toda a curva da pandemia não estavam disponíveis neste momento.

## Conclusão

A COVID-19 foi associada a uma redução significativa e abrupta de procedimentos diagnósticos cardíacos na América Latina, a qual foi mais relacionada às medidas de distanciamento social do que ao aumento da incidência da doença. São necessários melhor equilíbrio e timing de medidas de distanciamento social e planejamento para manter o acesso a atendimento médico geral e atendimento cardiovascular em particular durante um surto pandêmico, especialmente em regiões com alta mortalidade cardiovascular.

## *Material suplementar

Para informação adicional, por favor,clique aqui


