# Supplementary material for: O Impacto da COVID-19 no Diagnóstico de Doenças Cardíacas na América Latina Uma Subanálise do INCAPS COVID
Source: Arq Bras Cardiol. 2022 Jan 11;118(4):745–53. [Article in Portuguese] doi: 10.36660/abc.20210388 (PMC9007020; doi:10.36660/abc.20210388)
Supplement: Supplementary file 1 [file 2021-0388-supplemental-material.pdf]

Supplemental Material

INCAPS COVID

IAEA Noninvasive CArdiology Protocols Survey on the Impact of COVID-19 on Cardiovascular Diagnostic Testing Practices and Working Conditions

Appendix: Table of Contents

INCAPS COVID Investigators Group	Pages 2-15
Country Participation	Page 16
Survey Questions	Pages 17-21

INCAPS COVID Investigators Group

Executive Committee: Andrew J. Einstein (chair), Diana Paez (IAEA section head), Maurizio Dondi (IAEA project lead); (alphabetically) Nathan Better, Rodrigo Cerci, Sharmila Dorbala, Thomas N. B. Pascual, Paolo Raggi, Leslee J. Shaw, Todd C. Villines, Joao V. Vitola, Michelle C. Williams

Information Technology and Statistics Committee: Yaroslav Pynda (chair); (alphabetically): Gerd Hinterleitner, Yao Lu, Olga Morozova, Zhuoran Xu

Data Coordination Committee: Cole B. Hirschfeld (chair); (alphabetically): Yosef Cohen, Benjamin Goebel, Eli Malkovskiy, Michael Randazzo

Communications Committee: Andrew Choi (chair); (alphabetically): Juan Lopez-Mattei, Purvi Parwani

Members (alphabetically by country and last name)

Afghanistan: Mohammad Nawaz Nasery

Albania: Artan Goda, Ervina Shirka

Algeria: Rabie Benlabgaa, Salah Bouyoucef, Abdelkader Medjahedi, Qais Nailli

Argentina: Mariela Agolti, Roberto Nicolas Aguero, Maria del Carmen Alak, Lucia Graciela Alberguina, Guillermo Arroñada, Andrea Astesiano, Alfredo Astesiano, Carolina Bas Norton, Pablo Benteo, Juan Blanco, Juan Manuel Bonelli, Jose Javier Bustos, Raul Cabrejas, Jorge Cachero, Roxana Campisi, Alejandro Canderoli, Silvia Carames, Patrícia Carrascosa, Ricardo Castro, Oscar Cendoya, Luciano Martin Cognigni, Carlos Collaud, Carlos Collaud, Claudia Cortes, Javier Courtis, Daniel Cragnolino, Mariana Daicz, Alejandro De La Vega, Silvia Teresa De Maria, Horacio Del Riego, Fernando Dettori, Alejandro Deviggiano, Laura Dragonetti, Mario Embon, Ruben Emilio Enriquez,

Jorge Ensinas, Fernando Faccio, Adolfo Facello, Diego Garofalo, Ricardo Geronazzo, Natalia Gonza, Lucas Gutierrez, Miguel Angel Guzzo, Miguel Angel Guzzo, Victor Hasbani, Melina Huerin, Victor Jäger, Julio Manuel Lewkowicz, Maria Nieves A López De Munaín, Jose Maria Lotti, Alejandra Marquez, Osvaldo Masoli, Osvaldo Horacio Masoli, Edgardo Mastrovito, Matias Mayoraz, Graciela Eva Melado, Anibal Mele, Maria Fernanda Merani, Alejandro Horacio Meretta, Susana Molteni, Marcos Montecinos, Eduardo Noguera, Carlos Novoa, Claudio Pereyra Sueldo, Sebastian Perez Ascani, Pablo Pollono, Maria Paula Pujol, Alejandro Radzinschi, Gustavo Raimondi, Marcela Redruello, Marina Rodríguez, Matías Rodríguez, Romina Lorena Romero, Arturo Romero Acuña, Federico Rovaletti, Lucas San Miguel, Lucrecia Solari, Bruno Strada, Sonia Traverso, Sonia Simona Traverzo, Maria del Huerto Velazquez Espeche, Juan Sebastian Weihmuller, Juan Wolcan, Susana Zeffiro

Armenia: Mari Sakanyan

Australia: Scott Beuzeville, Raef Boktor, Patrick Butler, Jennifer Calcott, Loretta Carr, Virgil Chan, Charles Chao, Woon Chong, Mark Dobson, D'Arne Downie, Girish Dwivedi, Barry Elison, Jean Engela, Roslyn Francis, Anand Gaikwad, Ashok Gangasandra Basavaraj, Bruce Goodwin, Robert Greenough, Christian Hamilton-Craig, Victar Hsieh, Subodh Joshi, Karin Lederer, Kenneth Lee, Joseph Lee, John Magnussen, Nghi Mai, Gordon Mander, Fiona Murton, Dee Nandurkar, Johanne Neill, Edward O'Rourke, Patricia O'Sullivan, George Pandos, Kunthi Pathmaraj, Alexander Pitman, Rohan Poulter, Manuja Premaratne, David Prior, Lloyd Ridley, Natalie Rutherford, Hamid Salehi, Connor Saunders, Luke Scarlett, Sujith Seneviratne, Deepa Shetty, Ganesh Shrestha, Jonathan Shulman, Vijay Solanki, Tony Stanton, Murch Stuart, Michael Stubbs, Ian Swainson, Kim Taubman, Andrew Taylor, Paul Thomas, Steven Unger, Anthony Upton, Shankar Vamadevan, William Van Gaal, Johan Verjans, Demetrius Voutnis, Victor Wayne, Peter Wilson, David Wong, Kirby Wong, John Younger

Austria: Gudrun Feuchtner, Siroos Mirzaei, Konrad Weiss

Belarus: Natallia Maroz-Vadalazhskaya

Belgium: Olivier Gheysens, Filip Homans, Rodrigo Moreno-Reyes, Agnès Pasquet, Veronique Roelants, Caroline M. Van De Heyning

Bolivia: Raúl Araujo Ríos

Bosnia - Herzegovina: Valentina Soldat-Stankovic, Sinisa Stankovic

Brazil: Maria Helena Albernaz Siqueira, Augusto Almeida, Paulo Henrique Alves Togni, Jose Henrique Andrade, Luciana Andrade, Carlos Anselmi, Roberta Araújo, Guilherme Azevedo, Sabbrina Bezerra, Rodrigo Biancardi, Gabriel Blacher Grossman, Simone Brandão, Diego Bromfman Pianta, Lara Carreira, Bruno Castro, Tien Chang, Fernando Cunali Jr., Roberto Cury, Roberto Dantas, Fernando de Amorim Fernandes, Andrea De Lorenzo, Robson De Macedo Filho, Fernanda Erthal, Fabio Fernandes, Juliano Fernandes, Fabio Fernandes, Thiago Ferreira De Souza, Wilson Furlan Alves, Bruno Ghini, Luiz Goncalves, Ilan Gottlieb, Marcelo Hadlich, Vinícius Kameoka, Ronaldo Lima, Adna Lima, Rafael Willain Lopes, Ricardo Machado e Silva, Tiago Magalhães, Fábio Martins Silva, Luiz Eduardo Mastrocola, Fábio Medeiros, José Claudio Meneghetti, Vania Naue, Danilo Naves, Roberto Nolasco, Cesar Nomura, Joao Bruno Oliveira, Eduardo Paixao, Filipe Penna De Carvalho, Ibraim Pinto, Priscila Possetti, Mayra Quinta, Rodrigo Rizzo Nogueira Ramos, Ricardo Rocha, Alfredo Rodrigues, Carlos Rodrigues, Leila Romantini, Adelina Sanches, Sara Santana, Leonardo Sara da Silva, Paulo Schvartzman, Cristina Sebastião Matushita, Tiago Senra, Afonso Shiozaki, Maria Eduarda Menezes de Siqueira, Cristiano Siqueira, Paola Smanio, Carlos Eduardo Soares, José Soares Junior, Marcio Sommer Bittencourt, Bernardo Spiro, Cláudio Tinoco Mesquita, Jorge Torreao, Rafael Torres, Marly Uellendahl, Guilherme Urpia Monte, Otávia Veríssimo, Estevan Vieira Cabeda, Felipe Villela Pedras, Roberto Waltrick, Marcello Zapparoli

Brunei Darussalam: Hamid Naseer

Bulgaria: Marina Garcheva-Tsacheva, Irena Kostadinova

Cambodia: Youdaline Theng

Canada: Gad Abikhzer, Rene Barette, Benjamin Chow, Dominique Dabreo, Matthias Friedrich, Ria Garg, Mohammed Nassoh Hafez, Chris Johnson, Marla Kiess, Jonathon Leipsic, Eugene Leung, Robert Miller, Anastasia Oikonomou, Stephan Probst, Idan Roifman, Gary Small, Vikas Tandon, Adwait Trivedi, James White, Katherine Zukotynski

Chile: Jose Canessa, Gabriel Castro Muñoz, Carmen Concha, Pablo Hidalgo, Cesar Lovera, Teresa Massardo, Luis Salazar Vargas

Colombia: Pedro Abad, Harold Arturo, Sandra Ayala, Luis Benitez, Alberto Cadena, Carlos Caicedo, Antonio Calderón Moncayo, Antonio Calderón Moncayo, Sharon Gomez, Claudia T. Gutierrez Villamil, Claudia Jaimes, Juan Londoño, Juan Luis Londoño Blair, Luz Pabon, Mauricio Pineda, Juan Carlos Rojas, Diego Ruiz, Manuel Valencia Escobar, Andres Vasquez, Damiana Vergel, Alejandro Zuluaga

Costa Rica: Isabel Berrocal Gamboa, Gabriel Castro, Ulises González,

Croatia: Ana Baric, Tonci Batinic, Maja Franceschi, Maja Hrabak Paar, Mladen Jukic, Petar Medakovic, Viktor Persic, Marina Prpic, Ante Punda

Cuba: Juan Felipe Batista, Juan Manuel Gómez Lauchy, Yamile Marcos Gutierrez, Yamile Marcos Gutierrez, Rayner Menéndez, Amalia Peix, Luis Rochela

Cyprus: Christoforos Panagidis, Ioannis Petrou

Czech Republic: Vaclav Engelmann, Milan Kaminek, Vladimír Kincl, Otto Lang, Milan Simanek

Denmark: Jawdat Abdulla, Morten Bøttcher, Mette Christensen, Lars Christian Gormsen, Philip Hasbak, Søren Hess, Paw Holdgaard, Allan Johansen, Kasper Kyhl, Bjarne Linde Norgaard, Kristian Altern Øvrehus, Niels Peter Rønnow Sand, Rolf Steffensen, Anders Thomassen, Bo Zerahn

Dominican Republic: Alfredo Perez

Ecuador: Giovanni Alejandro Escorza Velez, Mayra Sanchez Velez

Egypt: Islam Shawky Abdel Aziz, Mahasen Abougabal, Taghreed Ahmed, Adel Allam, Ahmed Asfour, Mona Hassan, Alia Hassan, Ahmed Ibrahim, Sameh Kaffas, Ahmed Kandeel, Mohamed Mandour Ali, Ahmad Mansy, Hany Maurice, Sherif Nabil, Mahmoud Shaaban

El Salvador: Ana Camila Flores

Estonia: Anne Poksi

Finland: Juhani Knuuti, Velipekka Kokkonen, Martti Larikka, Valtteri Uusitalo

France: Matthieu Bailly, Samuel Burg, Jean-François Deux, Vincent Habouzit, Fabien Hyafil, Olivier Lairez, Franck Proffit, Hamza Regaieg, Laure Sarda-Mantel, Vania Tacher

Germany: Roman P. Schneider

Ghana: Harold Ayetey

Greece: George Angelidis, Aikaterini Archontaki, Sofia Chatziioannou, Ioannis Datseris, Christina Fragkaki, Panagiotis Georgoulias, Sophia Koukouraki, Maria Koutelou, Eleni Kyrozi, Evangelos Repasos, Petros Stavrou, Pipitsa Valsamaki

Guatemala: Carla Gonzalez, Goleat Gutierrez

Honduras: Alejandro Maldonado

Hungary: Klara Buga, Ildiko Garai, Pál Maurovich-Horvat, Erzsébet Schmidt, Balint Szilveszter, Edit Várady

India: Nilesh Banthia, Jinendra Kumar Bhagat, Rishi Bhargava, Vivek Bhat, Mona Bhatia, Partha Choudhury, Vijay Sai Chowdekar, Aparna Irodi, Shashank Jain, Elizabeth Joseph, Sukriti Kumar, Prof Dr Girijanandan Mahapatra, Deepanjan Mitra, Bhagwant Rai Mittal, Ahmad Ozair, Chetan Patel, Tapan Patel, Ravi Patel, Shivani Patel, Sudhir Saxena, Shantanu Sengupta, Santosh Singh, Bhanupriya Singh, Ashwani Sood, Atul Verma

Indonesia: Erwin Affandi, Padma Savenadia Alam, Edison Edison, Gani Gunawan, Habusari Hapkido, Basuki Hidayat, Aulia Huda, Anggoro Praja Mukti, Djoko Prawiro, Erwin Affandi Soeriadi, Hilman Syawaluddin

Iraq: Amjed Albadr

Islamic Republic of Iran: Majid Assadi, Farshad Emami, Golnaz Houshmand, Majid Maleki, Maryam Tajik Rostami, Seyed Rasoul Zakavi

Israel: Eed Abu Zaid, Svetlana Agranovich, Yoav Arnson, Rachel Bar-Shalom, Alex Frenkel, Galit Knafo, Rachel Lugassi, Israel Shlomo Maor Moalem, Maya Mor, Noam Muskal, Sara Ranser, Aryeh Shalev

Italy: Domenico Albano, Pierpaolo Alongi, Gaspare Arnone, Elisa Bagatin, Sergio Baldari, Matteo Bauckneht, Paolo Bertelli, Francesco Bianco, Rachele Bonfiglioli, Roberto Boni, Andrea Bruno, Isabella Bruno, Elena Busnardo, Elena Califaretti, Luca Camoni, Aldo Carnevale, Roberta Casoni, Armando Ugo Cavallo, Giorgio Cavenaghi, Franca Chierichetti, Marcello Chiocchi, Corrado Cittanti, Mauro Colletta, Umberto Conti, Alberto Cossu, Alberto Cuocolo, Marco Cuzzocrea, Maria Luisa De Rimini, Giuseppe De Vincentis, Eleonora Del Giudice, Alberico Del Torto, Veronica Della Tommasina, Rexhep Durmo, Paola Anna Erba, Laura Evangelista, Riccardo Faletti, Evelina Faragasso, Mohsen Farsad, Paola Ferro, Luigia Florimonte, Viviana Frantellizzi, Fabio Massimo Fringuelli, Marco Gatti, Angela Gaudiano, Alessia Gimelli, Raffaele Giubbini, Francesca Giuffrida, Salvatore Ialuna, Riccardo Laudicella, Lucia Leccisotti, Lucia Leva, Riccardo Liga, Carlo Liguori, Giampiero Longo, Margherita Maffione, Maria Elisabetta Mancini, Claudio Marcassa, Elisa Milan, Barbara Nardi, Sara Pacella, Giovanna Pepe, Gianluca Pontone, Sabina Pulizzi, Natale Quartuccio, Lucia Rampin, Fabrizio Ricci, Pierluigi Rossini, Giuseppe Rubini, Vincenzo Russo, Gian Mauro Sacchetti, Gianmario Sambuceti, Massimo Scarano, Roberto Sciagrà, Massimiliano Sperandio, Antonella Stefanelli, Guido Ventroni, Stefania Zoboli

Jamaica: Dainia Baugh, Duane Chambers, Ernest Madu, Felix Nunura

Japan: Hiroshi Asano, Chimura Misato Chimura, Shinichiro Fujimoto, Koichiro Fujisue, Tomohisa Fukunaga, Yoshimitsu Fukushima, Kae Fukuyama, Jun Hashimoto, Yasutaka Ichikawa, Nobuo Iguchi, Masamichi Imai, Anri Inaki, Hayato Ishimura, Satoshi Isobe, Toshiaki Kadokami, Takao Kato, Takashi Kudo, Shinichiro Kumita, Hirotaka Maruno, Hiroyuki Mataki, Masao Miyagawa, Ryota Morimoto, Masao Moroi, Shigeki Nagamachi, Kenichi Nakajima, Tomoaki Nakata, Ryo Nakazato, Mamoru Nanasato, Masanao Naya, Takashi Norikane, Yasutoshi Ohta, Satoshi Okayama, Atsutaka Okizaki, Yoichi Otomi, Hideki Otsuka, Masaki Saito, Sakata Yasushi Sakata, Masayoshi Sarai, Daisuke Sato, Shinya Shiraishi, Yoshinobu Suwa, Kentaro Takanami, Kazuya Takehana, Junichi Taki, Nagara Tamaki, Yasuyo Taniguchi, Hiroki Teragawa, Nobuo Tomizawa, Kenichi Tsujita, Kyoko Umeji, Yasushi Wakabayashi, Shinichiro Yamada, Shinya Yamazaki, Tatsuya Yoneyama

Jordan: Mohammad Rawashdeh

Kazakhstan: Daultai Batyrkhanov, Tairkhan Dautov

Kenya: Khalid Makhdomi, Kevin Ombati

Kuwait: Faridah Alkandari, Masoud Garashi

Lao People's Democratic Republic: Tchoyoson Lim Coie, Sonexay Rajvong

Latvia: Artem Kalinin, Marika Kalnina

Lebanon: Mohamad Haidar

Lithuania: Renata Komiagiene, Giedre Kviecinskiene, Mindaugas Mataciunas, Donatas Vajauskas

Luxembourg: Christian Picard

Malaysia: Noor Khairiah A. Karim

Malta: Luise Reichmuth, Anthony Samuel

Mauritius: Mohammad Aaftaab Allarakha, Ambedhkar Shantaram Naojee

Mexico: Erick Alexanderson-Rosas, Erika Barragan, Alejandro Becerril González-Montecinos, Manuel Cabada, Daniel Calderon Rodriguez, Isabel Carvajal-Juarez, Violeta Cortés, Filiberto Cortés, Erasmo De La Peña, Manlio Gama-Moreno, Luis González, Nelsy Gonzalez Ramírez, Moisés Jiménez-Santos, Luis Matos, Edgar Monroy, Martha Morelos, Mario Ornelas, Jose Alberto Ortga Ramirez, Andrés Preciado-Anaya, Óscar Ulises Preciado-Gutiérrez, Adriana Puente Barragan, Sandra Graciela Rosales Uvera, Sigelinda Sandoval, Miguel Santaularia Tomas, Lilia M. Sierra-Galan, Lilia M. Sierra-Galan, Silvia Siu, Enrique Vallejo, Mario Valles

Monaco: Marc Faraggi

Mongolia: Erdenechimeg Sereegotov

Montenegro: Srdja Ilic

Morocco: Nozha Ben-Rais, Nadia Ismaili Alaoui, Sara Taleb

Myanmar: Khin Pa Pa Myo, Phyo Si Thu

Nepal: Ram Kumar Ghimire, Bijoy Rajbanshi

Netherlands: Peter Barneveld, Andor Glaudemans, Jesse Habets, Klaas Pieter Koopmans, Jeroen Manders, Stefan Pool, Arthur Scholte, Asbjørn Scholtens, Riemer Slart, Paul Thimister, Erik-Jan Van Asperen, Niels Veltman, Derk Verschure, Nils Wagenaar

New Zealand: John Edmond, Chris Ellis, Kerryanne Johnson, Ross Keenan, Shaw Hua (Anthony) Kueh, Christopher Occleshaw, Alexander Sasse, Andrew To, Niels Van Pelt, Calum Young

Nicaragua: Teresa Cuadra, Hector Bladimir Roque Vanegas

Niger: Idrissa Adamou Soli, Djibrillou Moussa Issoufou

Nigeria: Tolulope Ayodele, Chibuzo Madu, Yetunde Onimode

Norway: Elen Efros-Monsen, Signe Helene Forsdahl, Jenni-Mari Hildre Dimmen, Arve Jørgensen, Isabel Krohn, Pål Løvhaugen, Anders Tjellaug Bråten

Oman: Humoud Al Dhuhli, Faiza Al Kindi, Naeema Al-Bulushi, Zabah Jawa, Naima Tag

Pakistan: Muhammad Shehzad Afzal, Shazia Fatima, Muhammad Numair Younis, Musab Riaz, Mohammad Saadullah

Panama: Yariela Herrera

Papua New Guinea: Dora Lenturut-Katal

Paraguay: Manuel Castillo Vázquez, José Ortellado

People's Republic of Bangladesh: Afroza Akhter

People's Republic of China: Dianbo Cao, Stephen Cheung, Xu Dai, Lianggeng Gong, Dan Han, Yang Hou, Caiying Li, Tao Li, Dong Li, Sijin Li, Jinkang Liu, Hui Liu, Bin Lu, Ming Yen Ng, Kai Sun, Gongshun Tang, Jian Wang, Ximing Wang, Zhao-Qian Wang, Yining Wang, Yifan Wang, Jiang Wu, Zhifang Wu, Liming Xia, Jiangxi Xiao, Lei Xu, Youyou Yang, Wu Yin, Jianqun Yu, Li Yuan, Tong Zhang, Longjiang Zhang, Yong-Gao Zhang, Xiaoli Zhang, Li Zhu

Peru: Ana Alfaro

Philippines: Paz Abrihan, Asela Barroso, Eric Cruz, Marie Rhiamar Gomez, Vincent Peter Magboo, John Michael Medina, Jerry Obaldo, Davidson Pastrana, Christian Michael Pawhay, Alvin Quinon, Jeanelle Margareth Tang, Bettina Tecson, Kristine Joy Uson, Mila Uy

Poland: Magdalena Kostkiewicz, Jolanta Kunikowska

Portugal: Nuno Bettencourt, Guilhermina Cantinho, Antonio Ferreira

Qatar: Ghulam Syed

Republic of Ireland: Samer Arnous, Said Atyani, Angela Byrne, Tadhg Gleeson, David Kerins, Conor Meehan, David Murphy, Mark Murphy, John Murray, Julie O'Brien

Republic of Korea: Ji-In Bang, Henry Bom, Sang-Geon Cho, Chae Moon Hong, Su Jin Jang, Yong Hyu Jeong, Won Jun Kang, Ji-Young Kim, Jaetae Lee, Chang Kyeong Namgung, Young So, Kyoung Sook Won

Republic of North Macedonia: Venjamin Majstorov, Marija Vavlukis

Republic of Slovenia: Barbara Gužic Salobir, Monika Štalc

Romania: Theodora Benedek, Imre Benedek, Raluca Mititelu, Claudiu Adrian Stan

Russian Federation: Alexey Ansheles, Olga Dariy, Olga Drozdova, Nina Gagarina, Vsevolod Milyevich Gulyaev, Irina Itskovich, Anatoly Karalkin, Alexander Kokov, Ekaterina Migunova, Viktor Pospelov, Daria Ryzhkova, Guzaliya Saifullina, Svetlana Sazonova, Vladimir Sergienko, Irina Shurupova, Tatjana Trifonova, Wladimir Yurievich Ussov, Margarita Vakhromeeva, Nailya Valiullina, Konstantin Zavadovsky, Kirill Zhuravlev

Saudi Arabia: Mirvat Alasnag, Subhani Okarvi

Serbia: Dragana Sobic Saranovic

Singapore: Felix Keng, Jia Hao Jason See, Ramkumar Sekar, Min Sen Yew

Slovak Republic: Andrej Vondrak

South Africa: Shereen Bejai, George Bennie, Ria Bester, Gerrit Engelbrecht, Osayande Evbuomwan, Harlem Gongxeka, Magritha Jv Vuuren, Mitchell Kaplan, Purbhoo Khushica, Hoosen Lakhi, Lizette Louw, Nico Malan, Katarina Milos, Moshe Modiselle, Stuart More, Mathava Naidoo, Leonie Scholtz, Mboyo Vangu

Spain: Santiago Aguadé-Bruix, Isabel Blanco, Antonio Cabrera, Alicia Camarero, Irene Casáns-Tormo, Hug Cuellar-Calabria, Albert Flotats, Maria Eugenia Fuentes Cañamero, María Elia García, Amelia Jimenez-Heffernan, Rubén Leta, Javier Lopez Diaz, Luis Lumbreras, Juan Javier Marquez-Cabeza, Francisco Martin, Anxo Martinez de Alegria, Francisco Medina, Maria Pedrera Canal,

Virginia Peiro, Virginia Pubul-Nuñez, Juan Ignacio Rayo Madrid, Cristina Rodríguez Rey, Ricardo Ruano Perez, Joaquín Ruiz, Gertrudis Sabatel Hernández, Ana Sevilla, Nahla Zeidán

Sri Lanka: Damayanthi Nanayakkara, Chandraguptha Udugama

Sweden: Magnus Simonsson

Switzerland: Hatem Alkadhi, Ronny Ralf Buechel, Peter Burger, Luca Ceriani, Bart De Boeck, Christoph Gräni, Alix Juillet de Saint Lager Lucas, Christel H. Kamani, Nadine Kawel-Boehm, Robert Manka, John O. Prior, Axel Rominger, Jean-Paul Vallée

Thailand: Benjapa Khiewvan, Teerapon Premprabha, Tanyaluck Thientunyakit

Tunisia: Ali Sellem

Turkey: Kemal Metin Kir, Haluk Sayman

Uganda: Mugisha Julius Sebikali, Zerida Muyinda

Ukraine: Yaroslav Kmetyuk, Pavlo Korol, Olena Mykhalchenko, Volodymyr Pliatsek, Maryna Satyr

United Arab Emirates: Batool Albalooshi, Mohamed Ismail Ahmed Hassan

United Kingdom: Jill Anderson, Punit Bedi, Thomas Biggans, Anda Bularga, Russell Bull, Rajesh Burgul, John-Paul Carpenter, Duncan Coles, David Cusack, Aparna Deshpande, John Dougan, Timothy Fairbairn, Alexia Farrugia, Deepa Gopalan, Alistair Gummow, Prasad Guntur Ramkumar, Mark Hamilton, Mark Harbinson, Thomas Hartley, Benjamin Hudson, Nikhil Joshi, Michael Kay, Andrew Kelion, Azhar Khokhar, Jamie Kitt, Ken Lee, Chen Low, Sze Mun Mak, Ntouskou Marousa, Jon Martin, Elisa Mcalindon, Leon Menezes, Gareth Morgan-Hughes, Alastair Moss, Anthony Murray, Edward Nicol, Dilip Patel, Charles Peebles, Francesca Pugliese, Jonathan Carl Luis Rodrigues, Christopher Rofe, Nikant Sabharwal, Rebecca Schofield, Thomas Semple, Naveen Sharma, Peter Strouhal, Deepak Subedi, William Topping, Katharine Tweed, Jonathan Weir-Mccall

United States of America: Suhny Abbara, Taimur Abbasi, Brian Abbott, Shady Abohashem, Sandra Abramson, Tarek Al-Abboud, Mouaz Al-Mallah, Omar Almousalli, Karthikeyan Ananthasubramaniam, Mohan Ashok Kumar, Jeffrey Askew, Lea Attanasio, Mallory Balmer-Swain, Richard R. Bayer, Adam Bernheim, Sabha Bhatti, Erik Bieging, Ron Blankstein, Stephen Bloom, Sean Blue, David Bluemke, Andressa Borges, Kelley Branch, Paco Bravo, Jessica Brothers, Matthew Budoff, Renée Bullock-Palmer, Angela Burandt, Floyd W. Burke, Kelvin Bush, Candace Candela, Elizabeth Capasso, Joao Cavalcante, Donald Chang, Saurav Chatterjee, Yiannis Chatzizisis, Michael Cheezum, Tiffany Chen, Jennifer Chen, Marcus Chen, Andrew Choi, James Clarcq, Ayreen Cordero, Matthew Crim, Sorin Danciu, Bruce Decter, Nimish Dhruva, Neil Doherty, Rami Doukky, Anjori Dunbar, William Duvall, Rachael Edwards, Kerry Esquitin, Husam Farah, Emilio Fentanes, Maros Ferencik, Daniel Fisher, Daniel Fitzpatrick, Cameron Foster, Tony Fuisz, Michael Gannon, Lori Gastner, Myron Gerson, Brian Ghoshhajra, Alan Goldberg, Brian Goldner, Jorge Gonzalez, Rosco Gore, Sandra Gracia-López, Fadi Hage, Agha Haider, Sofia Haider, Yasmin Hamirani, Karen Hassen, Mallory Hatfield, Carolyn Hawkins, Katie Hawthorne, Nicholas Heath, Robert Hendel, Phillip Hernandez, Gregory Hill, Stephen Horgan, Jeff Huffman, Lynne Hurwitz, Ami Iskandrian, Rajesh Janardhanan, Christine Jellis, Scott Jerome, Dinesh Kalra, Summanther Kaviratne, Fernando Kay, Faith Kelly, Omar Khalique, Mona Kinkhabwala, George Kinzfogl Iii, Jacqueline Kircher, Rachael Kirkbride, Michael Kontos, Anupama Kottam, Joseph Krepp, Jay Layer, Steven H Lee, Jeffrey Leppo, John Lesser, Steve Leung, Howard Lewin, Diana Litmanovich, Yiyan Liu, Juan Lopez-Mattei, Kathleen Magurany, Jeremy Markowitz, Amanda Marn, Stephen E Matis, Michael Mckenna, Tony Mcrae, Fernando Mendoza, Michael Merhige, David Min, Chanan Moffitt, Karen Moncher, Warren Moore, Shamil Morayati, Michael Morris, Mahmud Mossa-Basha, Zorana Mrsic, Venkatesh Murthy, Prashant Nagpal, Kyle Napier, Jagat Narula, Katarina Nelson, Prabhjot Nijjar, Medhat Osman, Purvi Parwani, Edward Passen, Amit Patel, Pravin Patil, Ryan Paul, Lawrence Phillips, Venkateshwar Polsani, Rajaram Poludasu, Brian Pomerantz, Thomas Porter, Ryan Prentice, Amit Pursnani, Mark Rabbat, Suresh Ramamurti, Florence Rich, Hiram Rivera Luna, Austin Robinson, Kim Robles, Cesar Rodríguez, Mark Rorie, John Rumberger, Raymond Russell, Philip Sabra, Diego Sadler, Mary Schemmer, U. Joseph Schoepf, Samir Shah, Nishant Shah, Sujata Shanbhag, Gaurav Sharma, Steven

Shayani, Jamshid Shirani, Pushpa Shivaram, Steven Sigman, Mitch Simon, Ahmad Slim, David Smith, Alexandra Smith, Prem Soman, Aditya Sood, Monvadi Barbara Srichai-Parsia, James Streeter, Albert T, Ahmed Tawakol, Dustin Thomas, Randall Thompson, Tara Torbet, Desiree Trinidad, Shawn Ullery, Samuel Unzek, Seth Uretsky, Srikanth Vallurupalli, Vikas Verma, Alfonso Waller, Ellen Wang, Parker Ward, Gaby Weissman, George Wesbey, Kelly White, David Winchester, David Wolinsky, Sandra Yost, Michael Zgaljardic

Uruguay: Omar Alonso, Mario Beretta, Rodolfo Ferrando, Miguel Kapitan, Fernando Mut

Uzbekistan: Omoa Djuraev, Gulnora Rozikhodjaeva

Vietnam: Ha Le Ngoc, Son Hong Mai, Xuan Canh Nguyen

Country Participation

We used IAEA-specific coding for world regions. Specifically, region participation included the following countries:

- **Africa** (n=11 countries): Algeria, Egypt, Ghana, Kenya, Mauritius, Morocco, Niger, Nigeria, South Africa, Tunisia, Uganda
- **Eastern Europe** (n=22 countries): Albania, Armenia, Belarus, Bosnia – Herzegovina, Bulgaria, Croatia, Czech Republic, Estonia, Hungary, Kazakhstan, Latvia, Lithuania, Montenegro, Poland, Republic of North Macedonia, Republic of Slovenia, Romania, Russian Federation, Serbia, Slovak Republic, Ukraine, Uzbekistan
- **Far East** (n=8 countries): Cambodia, Japan, Lao People's Democratic Republic, Mongolia, People's Republic of China, Philippines, Republic of Korea, Vietnam
- **Latin America** (n=19 countries): Argentina, Bolivia, Brazil, Chile, Colombia, Costa Rica, Cuba, Dominican Republic, Ecuador, El Salvador, Guatemala, Honduras, Jamaica, Mexico, Nicaragua, Panama, Paraguay, Peru, Uruguay
- **Middle East and South Asia** (n=16): Afghanistan, India, Iraq, Islamic Republic of Iran, Israel, Jordan, Kuwait, Lebanon, Nepal, Oman, Pakistan, People's Republic of Bangladesh, Qatar, Saudi Arabia, Sri Lanka, United Arab Emirates
- **North America** (n=2 countries): Canada, United States of America
- **South East Asia and the Pacific** (n=9 countries): Australia, Brunei Darussalam, Indonesia, Malaysia, Myanmar, New Zealand, Papua New Guinea, Singapore, Thailand
- **Western Europe** (n=21 countries): Austria, Belgium, Cyprus, Denmark, Finland, France, Germany, Greece, Italy, Luxembourg, Malta, Monaco, Netherlands, Norway, Portugal, Republic of Ireland, Spain, Sweden, Switzerland, Turkey, United Kingdom

Survey Questions

INCAPS COVID: IAEA Noninvasive CArdiology Protocols Survey on the impact of COVID-19 on cardiac imaging practices and working conditions

Kindly complete no more than one survey from each laboratory/centre. We ask individuals within each centre to talk with their colleagues and share the information so that only one survey is completed per facility. If you are affiliated with multiple laboratories/centres, we would be grateful if you would complete the survey separately for each. The deadline for completing the survey has been extended until May 30.

**About Yourself**	
* Your First/Given Name (Example: Diana)	
* Your Family Name/Surname (Example: Paez)	
Your Title (example: MD, PhD)	
* Would you like to be listed as a member of INCAPS COVID Investigators Group for publications?	☐ Yes ☐ No
* Your Email	
Your Telephone Number (include country code)	
* Your Profession (choose one, reflecting your major specialty)	☐ Cardiologist<br>☐ Radiologist<br>☐ Nuclear Medicine Physician<br>☐ Technologist/Radiographer<br>☐ Nurse Practitioner<br>☐ Physician Assistant<br>☐ Physicist<br>☐ Other
* Cardiac modaliti(es) practiced	☐ CT<br>☐ SPECT<br>☐ PET<br>☐ Echocardiography<br>☐ MRI
Number of years post-training performing cardiac imaging	
* Redeployment outside of usual imaging service(s) during the past few months (select all that apply)	☐ Yes ☐ No
Please specify	☐ Fever Clinic<br>☐ Emergency Department<br>☐ Inpatient COVID-19 service<br>☐ Inpatient General service<br>☐ Intensive Care Unit<br>☐ Workforce/Occupational Health<br>☐ Other

About second individual who contributed data to this survey	
* Is there a second individual who contributed data to this survey and would like to be listed as a member of INCAPS COVID Investigators Group for publications?	☐ Yes ☐ No
Their First/Given Name (Example: Rodrigo)	
Their Family Name/Surname (Example: Cerci)	
Their Title(s) (Example: MD)	

Page 2

**About Your Facility**	
* Name of Facility	
* Type of Site	☐ Hospital Inpatient<br>☐ Hospital Mixed Inpatient/Outpatient<br>☐ Hospital Outpatient<br>☐ Outpatient Facility With Physician Practices<br>☐ Outpatient Imaging-only Facility<br>☐ Emergency Department<br>☐ Observation Unit
* University-Affiliated Teaching Facility?	☐ Yes ☐ No
Number of beds (if Hospital):	
**Address**	
* *Country/Administrative Area*	
Province/State/Territory	
* *City*	
Zip Code/Postal Code	

At any time during the pandemic, has the institution's supply of the following materials been insufficient to meet the needs of cardiac imaging staff and patients?

	**Always Been Sufficient**	**At Times Insufficient**	**Never Available**
Surgical Masks	☐	☐	☐
N95/KN95/KF94/FFP2 Masks	☐	☐	☐
Gloves	☐	☐	☐
Gowns	☐	☐	☐
Eye Shielding	☐	☐	☐
Technetium-99m Generators	☐	☐	☐
F-18 FDG	☐	☐	☐
N-13 Ammonia	☐	☐	☐
Rubidium (Rb-82) Generators	☐	☐	☐
Cold kits (Sestamibi/Tetrofosmin)	☐	☐	☐

Page 3

Laboratory/Facility	
**For each of the following activities, with reference to nuclear cardiology and/or cardiac CT, please select whether your laboratory or facility does not currently have plans to implement it (N), is planning (P) to implement it but has not done so yet, or has already implemented (I) it:**	
Some outpatient activities cancelled/postponed during peak pandemic	☐ No Plans ☐ Planning ☐ Implemented
All non-urgent outpatient activities cancelled/postponed during peak pandemic	☐ No Plans ☐ Planning ☐ Implemented
All outpatient activities cancelled/postponed during peak pandemic	☐ No Plans ☐ Planning ☐ Implemented
Phased re-opening/resumption of activities after peak pandemic	☐ No Plans ☐ Planning ☐ Implemented
Extended hours after peak pandemic	☐ No Plans ☐ Planning ☐ Implemented
New weekend hours after peak pandemic	☐ No Plans ☐ Planning ☐ Implemented
Use telehealth to maintain contact with patients	☐ No Plans ☐ Planning ☐ Implemented
Use telehealth for patient interactions aspects (registration, consent)	☐ No Plans ☐ Planning ☐ Implemented
Use (telehealth) remote reading/reporting/communication of studies	☐ No Plans ☐ Planning ☐ Implemented

Page 4

Patients/Visitors	
**For each of the following activities, with reference to nuclear cardiology and/or cardiac CT, please select whether your laboratory or facility does not currently have plans to implement it (N), is planning (P) to implement it but has not done so yet, or has already implemented (I) it:**	
Alteration in patient transport, e.g. spacing use of elevators	☐ No Plans ☐ Planning ☐ Implemented
Change waiting areas to allow physical distancing	☐ No Plans ☐ Planning ☐ Implemented
Separate spaces for patients with and without COVID-19	☐ No Plans ☐ Planning ☐ Implemented
Reducing patient time in waiting room	☐ No Plans ☐ Planning ☐ Implemented
Limit accompanying family members and/or visitors	☐ No Plans ☐ Planning ☐ Implemented
Take temperature measurements for all patients/visitors	☐ No Plans ☐ Planning ☐ Implemented

Administer a screening questionnaire to all patients/visitors	☐ No Plans ☐ Planning ☐ Implemented
Test for COVID19 in all patients prior to diagnostic testing	☐ No Plans ☐ Planning ☐ Implemented
Require cloth/surgical mask for all patients/visitors	☐ No Plans ☐ Planning ☐ Implemented

Page 5

**Testing protocols**	
**For each of the following activities, with reference to nuclear cardiology and/or cardiac CT, please select whether your laboratory or facility does not currently have plans to implement it (N), is planning (P) to implement it but has not done so yet, or has already implemented (I) it:**	
Avoid exercise stress testing, use pharmacologic testing if possible	☐ No Plans ☐ Planning ☐ Implemented
Modify cardiac nuclear imaging protocols (e.g. higher dose, shorter acquisition time, stress first when applicable)	☐ No Plans ☐ Planning ☐ Implemented
Modify cardiac CT protocols (e.g. less use of IV rate-control agents)	☐ No Plans ☐ Planning ☐ Implemented
Allocate additional time for each scan to allow for thorough cleaning/disinfection between patients	☐ No Plans ☐ Planning ☐ Implemented

Page 6

Staff Conditions	
**For each of the following activities, with reference to nuclear cardiology and/or cardiac CT, please select whether your laboratory or facility does not currently have plans to implement it (N), is planning (P) to implement it but has not done so yet, or has already implemented (I) it:**	
Limiting staff proximity to patients	☐ No Plans ☐ Planning ☐ Implemented
Mandate personal protective equipment	☐ No Plans ☐ Planning ☐ Implemented
Altering or eliminating protocols requiring close patient contact for extended time	☐ No Plans ☐ Planning ☐ Implemented
Rotating staff work shifts	☐ No Plans ☐ Planning ☐ Implemented

Page 7

Employment
**For each of the following activities, with reference to nuclear cardiology and/or cardiac CT, please select whether your laboratory or facility does not currently have plans to**

implement it (N), is planning (P) to implement it but has not done so yet, or has already implemented (I) it:	
Temporarily furloughed (placed on unpaid leave) some imaging physicians due to economic crisis from COVID-19	☐ No Plans ☐ Planning ☐ Implemented
Temporarily furloughed (placed on unpaid leave) some imaging staff due to economic crisis from COVID-19	☐ No Plans ☐ Planning ☐ Implemented
Reduced salaries of some imaging physicians due to economic crisis from COVID-19	☐ No Plans ☐ Planning ☐ Implemented
Reduced salaries of some imaging staff due to economic crisis from COVID-19 Laid off/fired some imaging physicians due to economic crisis from COVID-19	☐ No Plans ☐ Planning ☐ Implemented
Laid off/fired some imaging staff due to economic crisis from COVID-19	☐ No Plans ☐ Planning ☐ Implemented

Page 8

Procedure Number Estimates			
**This is the last page of the survey, and the most important data being collected. Please estimate the number of procedures in each category performed in your facility for each of the following months. Kindly speak with colleagues in these areas if you do not have sufficient expertise to estimate with reasonable accuracy. If you are unable to obtain the information, you can leave a cell blank. Thank you for your help in completing the survey.**			
	**March 2019**	**March 2020**	**April 2020**
Stress ECG (treadmill or bicycle, no imaging)			
Stress Echocardiogram			
Stress SPECT Myocardial Perfusion Imaging			
Stress PET Myocardial Perfusion Imaging			
Stress Cardiac MRI			
CT Coronary Artery Calcium Scoring Alone			
CT Coronary Angiography (with or without Calcium Scoring)			
Transthoracic Echocardiogram (TTE, rest)			
Transesophageal Echocardiogram (TEE)			
PET for Endocarditis Evaluation			
Cardiac MRI (without stress)			
Invasive Coronary Angiography			
