# Supplementary material for: O Impacto da COVID-19 no Diagnóstico de Doenças Cardíacas na América Latina Uma Subanálise do INCAPS COVID
Source: Arq Bras Cardiol. 2022 Jan 11;118(4):745–53. [Article in Portuguese] doi: 10.36660/abc.20210388 (PMC9007020; doi:10.36660/abc.20210388)
Supplement: Translation

# Translation: The Impact of COVID-19 on Diagnosis of Heart Disease in Latin America an INCAPS COVID Sub-analysis

**Authors:** Rodrigo Julio Cerci, João Vicente Vitola, Diana Paez, Alejandro Zuluaga, Marcio Sommer Bittencourt, Lilia M. Sierra-Galan, Patricia Carrascosa, Roxana Campisi, Claudia Gutierrez-Villamil, Amalia Peix, Duane Chambers, Mayra Sánches Velez, Carla M. G. Alvarado, Ana C. F. Ventura, Alejandro Maldonado, Alfredo P. Castanos, Teresa C. Diaz, Yariela Herrera, Manuel C. Vasquez, Ana A. Arrieta, Fernando Mut, Cole Hirschfeld, Eli Malkovskiy, Benjamin Goebel, Yosef Cohen, Michael Randazzo, Leslee J. Shaw, Michelle C. Williams, Todd C. Villines, Nathan Better, Sharmila Dorbala, Paolo Raggi, Thomas N. B. Pascual, Yaroslav Pynda, Maurizio Dondi, Andrew J. Einstein

**Affiliations:** 1 Brasil Quanta Diagnóstico por Imagem - Cardiovascular CT, Curitiba, PR - Brasil; 2 Áustria International Atomic Energy Agency - Division of Human Health, Vienna - Áustria; 3 Colômbia Cedimed Quirosalud, Universidad Pontificia Bolivariana y Universidad CES, Medelin - Colômbia; 4 Brasil Diagnósticos da América SA – DASA, Barueri, SP - Brasil; 5 México American British Cowdray Medical Center IAP, Mexico City, Mexico City - México; 6 Argentina Diagnostico Maipu, Buenos Aires - Argentina; 7 Argentina Instituto Argentino de Diagnostico y Tratamiento S.A, Buenos Aires - Argentina; 8 Colômbia Fundación Cardioinfantil-Instituto de Cardiologia, Bogota - Colômbia; 9 Cuba Institute of Cardiology and Cardiovascular Surgery - Department of Nuclear Medicine, La Habana - Cuba; 10 Jamaica Radiology West, Montego Bay - Jamaica; 11 Equador Carlos Andrade Marin Specialty Hospital, Quito - Equador; 12 Guatemala Tecnodiagnosis, Guatemala - Guatemala; 13 El Salvador Radiology Clinic Brito Mejia Pena, San Salvador - El Salvador; 14 Honduras Servicio de Medicina Nuclear, Tegucigalpa - Honduras; 15 República Dominicana Centro Diagnostico Especializado, Santo Domingo - República Dominicana; 16 Panamá Hospital Salud Integral, Panama City - Panamá; 17 Panamá Hospital Santo Tomas - Nuclear Medicine Section, Panama - Panamá; 18 Paraguai Hospital Central, Instituto de Prevision Social, Asuncion - Paraguai; 19 Costa Rica Hospital Dr. Rafael Angel Calderon Guardia, San Jose - Costa Rica; 20 Uruguai Italian Hospital - Nuclear Medicine Service, Montevideo - Uruguai; 21 Estados Unidos da América Columbia University Irving Medical Center and New York-Presbyterian Hospital - Department of Medicine, New York - Estados Unidos da América; 22 Estados Unidos da América Seymour, Paul and Gloria Milstein Division of Cardiology, New York - Estados Unidos da América; 23 Estados Unidos da América Weill Cornell Medical College and New York-Presbyterian Hospital,New York - Estados Unidos da América; 24 Israel Technion Israel Institute of Technology, Haifa - Israel; 25 Reino Unido da Grã-Bretanha BHF Centre for Cardiovascular Science, University of Edinburgh, Edinburg - Reino Unido da Grã-Bretanha; 26 Estados Unidos da América University of Virginia, Charlottesville, Virginia - Estados Unidos da América; 27 Austrália Royal Melbourne Hospital and University of Melbourne, Melbourne - Austrália; 28 Estados Unidos da América Brigham and Women’s Hospital, Boston, Massachusetts - Estados Unidos da América; 29 Canadá University of Alberta - Department of Medicine and Division of Cardiology, Edmonton, Alberta - Canadá; 30 Filipinas Philipines Nuclear Research Institute, Manila - Filipinas; 31 Áustria International Atomic Energy Agency - Division of Human Health, Vienna - Áustria

**Keywords:** Cardiac Testing, Coronavírus, COVID-19, Cardiovascular Disease, Global Health

## Abstract

**Background:**

The COVID-19 pandemic has disrupted the delivery of care for cardiovascular diseases in Latin America. However, the effect of the pandemic on the cardiac diagnostic procedure volumes has not been quantified.

**Objective:**

To assess (1) the impact of COVID-19 on cardiac diagnostic volumes in Latin America and (2) determine its relationship with COVID-19 case incidence and social distancing measures.

**Methods:**

The International Atomic Energy Agency conducted a worldwide survey assessing changes in cardiac diagnostic volumes resulting from COVID-19. Cardiac diagnostic volumes were obtained from participating sites for March and April 2020 and compared to March 2019. Social distancing data were collected from Google COVID-19 community mobility reports and COVID-19 incidence per country from the Our World in Data.

**Results:**

Surveys were conducted in 194 centers performing cardiac diagnostic procedures, in 19 countries in Latin America. Procedure volumes decreased 36% from March 2019 to March 2020, and 82% from March 2019 to April 2020. The greatest decreases occurred in echocardiogram stress tests (91%), exercise treadmill tests (88%), and computed tomography calcium scores (87%), with slight variations between sub-regions of Latin America. Changes in social distancing patterns (p < 0.001) were more strongly associated with volume reduction than COVID-19 incidence (p = 0.003).

**Conclusions:**

COVID-19 was associated with a significant reduction in cardiac diagnostic procedures in Latin America, which was more related to social distancing than to the COVID-19 incidence. Better balance and timing of social distancing measures and planning to maintain access to medical care is warranted during a pandemic surge, especially in regions with high cardiovascular mortality.

## Summary statement

The COVID-19 pandemic was associated with a significant reduction in cardiac diagnostic procedures in Latin America in April 2020, which was more related to social distancing measures than to the COVID-19 incidence.

## Key points

- Cardiac diagnostic procedure volumes decreased 36% from March 2019 to March 2020, and 82% from March 2019 to April 2020.
- Changes in social distancing patterns (p < 0.001) were more strongly associated with volume reduction than COVID-19 incidence (p = 0.003).
- A better timing of social distancing measures and planning to maintain access to medical care is warranted during a pandemic surge, especially in high cardiovascular mortality regions.

## Introduction

Cardiovascular disease (CVD) remains the leading cause of mortality worldwide, including in Latin America (LATAM).^1 , 2^While mortality rates have progressively decreased over the past four decades in most high-income countries, the same phenomenon has not been observed in low- and middle-income countries, many of them in the LATAM region.^3^

A comprehensive approach to managing CVD and reducing associated mortality involves adequate prevention, including control of risk factors, appropriate use of tests to diagnose and guide treatment, and the establishment of appropriate therapies. The World Health Organization recently called attention to the worldwide disruption of healthcare caused by the COVID-19 pandemic, which unfortunately imposes an additional burden to CVD patient care in regions such as LATAM that have been severely affected by COVID-19.^4^

The International Atomic Energy Agency (IAEA) Division of Human Health aims to support member states to combat CVD, cancer, malnutrition, and other diseases through the use of appropriate prevention, diagnostic testing and treatment. Accordingly, the IAEA coordinated a worldwide survey of cardiovascular imaging centers (the IAEA Noninvasive Cardiology Protocols Study of COVID-19, INCAPS COVID survey), in order to assess the impact of the pandemic on the diagnostic evaluation of CVD.

The objectives of this study were: (1) to assess the impact of COVID-19 on cardiac diagnostic procedural volumes in LATAM and (2) to determine its relationship with COVID-19 case incidence, temporal presentation, and social distancing interventions. Understanding the relationship of the pandemic phases, social distancing measures, and the provision of CVD diagnosis in LATAM is crucial to being better prepared for similar situations in the future.

## Methods

### Study design

Data for this study were collected as part of the IAEA survey on the impact of COVID-19 on cardiac imaging (INCAPS COVID) and correlated with publicly available social distancing metrics from Google Community Mobility Reports and monthly COVID-19 incidence from Our World in Data database in LATAM.^5 - 7^The INCAPS COVID survey included questions regarding the healthcare facility, healthcare professionals, personal protective equipment, strategic plans for reopening, and changes in procedural volumes for a range of cardiovascular diagnostic procedures (Appendix).

### Data collection

Based on the IAEA standardized methodology, an electronic data entry system was designed to collect data, employing a secure software platform, the International Research Integration System (IRIS, https://iris.iaea.org). In INCAPS COVID no patient-specific or confidential data were collected, and participation by study sites was voluntary; therefore, no external ethics committee review was required.

Participants were asked to provide estimates of cardiac diagnostic procedure volumes from March 2019, March 2020, and April 2020, including the following: transthoracic and transesophageal echocardiography, cardiac magnetic resonance (CMR), stress testing (exercise treadmill test, echocardiography stress test, single-photon emission computed tomography [SPECT], positron emission tomography [PET], and CMR), PET infection studies, computed tomography calcium score, coronary computed tomography angiography, and invasive coronary angiography. For analysis purposes, we divided LATAM into the following sub-regions: South America (Argentina, Bolivia, Brazil, Chile, Colombia, Ecuador, Paraguay, Peru, and Uruguay); Central America and Mexico (Costa Rica, Guatemala, Honduras, Mexico, Nicaragua, Panama, and El Salvador); and The Caribbean (Cuba, Dominican Republic, and Jamaica).

### COVID-19 cases by country

Numbers of COVID-19 cases for each LATAM country were downloaded from the open access website Our World in Data (https://ourworldindata.org/coronavirus-source-data), which collects data from different official sources worldwide.^5^Our World in Data is a collaborative effort between researchers at the University of Oxford based at the Oxford Martin Programme on Global Development, who are the scientific editors of the website content, and the non-profit organization Global Change Data Lab, which publishes and maintains the website and the data tools. Data collected ranged from February 2020 to July 2020 to better reflect the pandemic evolution in LATAM. The number of new cases per million inhabitants per month per country was used for analysis.

### Mobility data by country

Mobility data was downloaded from Google Community Mobility Reports (https://www.google.com/covid19/mobility/) that collect mobility trends in 6 different categories: retail and recreation, grocery and pharmacy, parks, transit stations, workplaces, and residential. The baseline was the median value, for the corresponding day of the week, during the 5-week period January 3 to February 6, 2020.^6^Google calculates these insights based on data from users who have opted in to location history for their Google account, so the data represents a sample of all users. We used the change in time spent at home (residential) by month as an “immobility index” variable, from February 2020 to July 2020, which reflects changes in social distancing patterns during that period of time. Mobility data from Cuba was not available and, therefore, was not used in these analyses.

### Statistical analysis

Survey question responses are presented as numbers and percentages. Total procedures per center are presented as median and interquartile range. Percentage change in procedure volume was compared between March 2019 and March or April 2020 using the nonparametric Kruskal-Wallis test with asymptotic two-sided p values. In order to evaluate the association of the changes in cardiac procedure volumes with changes in mobility and COVID-19 incidence, we built a generalized estimating equation using countries as individual units and the monthly exam numbers as the outcome with month as a time variable. Statistical analysis was performed in Stata (version 15.1, Stata Corporation, LLC, College Station, Texas), Microsoft Excel (2016), and choropleth maps were constructed in R (version 4.0.1, R Development Core Team, Vienna, Austria) using tmap and rnaturalearth packages.

## Results

### Centers and procedure reduction

Data were obtained from 194 inpatient and outpatient centers in 19 countries in LATAM. The larger regional countries Brazil, Argentina, and Mexico were also the countries that contributed data from the largest number of centers: 70, 54, and 23 centers respectively. Characteristics of all centers are summarized in Table 1 . A total of 198,597 cardiac diagnostic procedures were performed at participating sites during the three months considered.

**Table 1:** – Characteristics of centers in Latin America

	South America	Central America and Mexico	The Caribbean
**Countries**	**9**	**7**	**3**
**Number of centers**	**155**	**31**	**8**
**Teaching institution (n, %)**	**69 (44.5)**	**21 (67.7)**	**4 (50)**
**Hospital beds (median, IQR)**	**202.5 (120 - 400)**	**167 (100 - 300)**	**168 (80 – 412.5)**
**Type of institution (n, %)**			
Hospital, inpatient only	3 (1.9)	5 (16.1)	0 (0)
Hospital, outpatient only	3 (1.9)	0(0)	0 (0)
Hospital, inpatient and outpatient	91 (58.7)	19 (61.3)	4 (50)
Outpatient imaging center	45 (29.0)	2 (6.4)	2 (25)
Outpatient physician practice	13 (8.4)	5 (16.1)	2 (25)
**Total procedures per center (median, IQR)**			
March 2019	157 (67 - 502)	91 (38 - 430)	173 (53.5 - 559.5)
March 2020	89 (31 - 253)	35 (19 - 143)	147.5 (24.5 - 343.5)
April 2020	32 (7 - 97)	20 (1 - 53)	36 (2.5 - 117)
**% Reduction from March 2019 to April 2020**			
Transthoracic echocardiogram	80.1	54.0	87.0
Transesophageal echocardiogram	81.6	88.0	89.6
Cardiac magnetic resonance	77.2	80.9	100
Computer tomography calcium score	81.7	96.1	99.5
Coronary computed tomography	73.0	84.8	85.9
Invasive coronary angiography	63.8	77.7	70.8
Exercise treadmill test	88.4	84.9	95.6
Echo stress test	91.1	94.5	76.9
SPECT	84.0	81.0	97.8
PET	62.0	90.9	NA
Stress cardiac magnetic resonance	76.3	89.2	NA

*IQR: interquartile range; PET: positron emission tomography; SPECT: single-photon emission computed tomography.*

In LATAM, cardiac diagnostic procedure volumes decreased by 36% from March 2019 to March 2020, and 82% from March 2019 to April 2020 ( Figure 1 – Map). There was some variation between LATAM regions and by type of cardiac procedure, with the greatest decreases from March 2019 to April 2020 in echocardiography stress test (91%), exercise treadmill test (88%), and computed tomography calcium score (87%) ( Table 1 ). The smallest decreases were reported for invasive coronary angiography (67%) and cardiac PET (65%). Procedure volumes also markedly decreased from March 2020 to April 2020. These decreases were significant (p < 0.001) in combination ( Figure 2 ) and for each procedure. Separate generalized linear models for LATAM regions and overall found significant declines in procedural volume (p < 0.001), using regression models weighted by 2019 procedural volume. In the 194 facilities in our study, an estimated 129,030 cardiac diagnostic procedures, which would have been performed based on the procedure rates from March 2019, were not performed during these two months of the pandemic.

**Figure 1:** – Color-coded map of Latin America displaying the reduction in total cardiac diagnostic procedure volumes by country from March 2019 to April 2020, in the beginning of the COVID-19 pandemic.

**Figure 2:** – Reduction in total cardiac diagnostic procedure volumes in sub-regions of Latin America: South America; Central America and Mexico; and The Caribbean from March 2019 to March 2020 and April 2020, in the beginning of the COVID-19 pandemic.

### Mobility data, COVID-19 incidence, and reduction in procedure volume

The greatest increase in time spent at home (immobility index) occurred during the month of April 2020 in most countries. There was a mean 26.7% increase in April that dropped to 18.8% in July, when compared to baseline. Exceptions were Nicaragua and Chile, where immobility increased until June ( Figure 3 ).

**Figure 3:** – Right panel: Number of new COVID-19 cases per million people per month in 19 countries in Latin America. Left panel: Change in time spent at home (“immobility index”) per month, using the 5-week period from January 3 to February 6, 2020 as the baseline in 18 countries in Latin America (not including Cuba). Note that the greatest immobility occurred in April 2020 in most countries, in concordance with the abrupt drop in cardiac diagnostic procedures. On the other hand, the number of new COVID-19 cases per million people was still progressively rising from March to July 2020 in most countries.

However, the COVID-19 pandemic was still in its early stages in most countries in April 2020, with a total of 199,277 cases by the end of the month. In most countries, the new monthly COVID-19 cases per million inhabitants continued rising and had not yet peaked by July 2020 ( Figure 3 ). Exceptions were Cuba and Jamaica, which peaked in April. By the end of July the number of cases rose 23.5 times, totaling 4,681,377 confirmed COVID-19 cases.^5^

Both the reduction in mobility and the increase in COVID-19 incidence were associated with the reduction in cardiac diagnostic procedures in the generalized models (p < 0.001). When fitting a multivariable model, mobility (p < 0.001) was more strongly associated with volume reduction than COVID-19 incidence (p = 0.003).

## Discussion

The results show a significant decrease in the number of cardiac diagnostic procedures performed in LATAM during the COVID-19 pandemic. The greatest decrease in the number of diagnostic procedures occurred in the month with the lowest mobility (April), which in turn coincided with the strictest quarantine periods in each country.

In most LATAM countries, social isolation was introduced in March, even without a significant number of COVID-19 cases. In March and April, there was a greater decrease in the number of cardiac diagnostic procedures conducted in LATAM, compared to Western Europe (46% decrease in March and 69% in April) and to US/Canada (39% decrease in March and 68% in April), despite the fact that these regions were experiencing the first peak of the pandemic, whereas LATAM was barely in the early stages.^7^By the end of March, fewer than 250 cases had been reported in 9 countries in the region, and, since that time, the number of cases has continued to increase. By late August 2020, SARS-CoV-2 had compromised all LATAM countries, with 7.15 million people affected.^5^The real number of cases may be even higher, since the number of tests per million remains low.^8 , 9^

Beyond the direct mortality caused by COVID-19, there were growing concerns regarding the consequences of the COVID-19 pandemic on health systems.^10 - 12^Fear of contagion in hospitals and health centers could have led to reluctance by patients to undergo cardiac diagnostic procedures. Additionally, elective interventions and consultations had to be postponed to prioritize COVID-19-related issues and avoid exposing patients to an unnecessary risk of infection in hospital or outpatient settings.^13^

LATAM frequently faces health problems that primarily affect the poor, added to health systems that are already fragile.^14 , 15^In such conditions, social distancing slows the peaking of the pandemic to allow countries with limited health resources to prepare for diagnosis and treatment of critically ill patients.^16 , 17^Nevertheless, according to Walker and colleagues, the LATAM countries that had the earliest first peak of the pandemic (Ecuador, Mexico, Brazil, Chile, Bolivia, Panama, Peru) or with unmitigated/limited health curves (Peru, Chile, Mexico, Ecuador) registered a higher death rate per million inhabitants.^18^This could be explained by the inability of these countries to prepare for the peak of the pandemic, finding their health systems vulnerable and producing high mortality figures. Similar events took place in European countries hit by the first wave of the COVID-19 pandemic, such as Italy and Spain.

Globally, approximately 70% of CVD deaths take place in low- and middle-income countries.^3 , 19^The long time interval between the start of social distancing measures and the first peak of the pandemic in LATAM countries limited patients’ access to cardiac diagnostic procedures, further delaying diagnosis and timely treatment of CVD. In the LATAM population, this cascade of events may increase cardiovascular morbidity and mortality, as has been reported in Brazil.^20^This survey was not designed to collect outcome information, but the negative effect of diagnostic delays will likely be corroborated in future studies for this purpose. A similar conclusion was reached by the Latin American Society of Interventional Cardiology when surveying the practice of Interventional Cardiology during the COVID-19 pandemic, focusing on myocardial infarction.^21^They reported a 51.2% reduction in care for ST-elevation myocardial infarction (STEMI), with the risk of increased mortality and/or morbidity following STEMI.

COVID-19 infection may be associated with cardiovascular events or mimic heart disease.^22 - 27^It is therefore essential, during the COVID-19 pandemic, to maintain the availability of all cardiac diagnostic modalities, in patients who are positive or negative for COVID-19.

There are several lessons we have learned for the future. We highlight five: 1) During a pandemic, access to cardiac diagnostic procedures should be maintained as much as possible for the entire population, regardless of the type of mobility restriction established in each country, strictly following the proper sanitary precautions. 2) Education campaigns should be established in the media and on social networks to explain to the community the importance of seeking help quickly in the face of warning signs of heart disease, while implementing measures to prevent the spread of COVID-19. 3) Non-COVID areas (“blue”) for the care of non-COVID pathologies and COVID areas (“red”) for infected patients should be established in health services. 4) It is necessary to guarantee worldwide access to health care supplies, from personal protective equipment to radiotracers.^28^5) Governments should guarantee health services not only for COVID patients but also for non-COVID patients with CVD.

### Limitations

The INCAPS COVID-19 survey evaluated data for the months of March and April 2020, when the pandemic was still in the initial phase in most LATAM countries. However, according to the evolution of lockdowns, the dates of economic reopening, and the mobility data for each country, April 2020 was the month with the least activity in these countries. The survey was carried out in a limited number of hospitals and diagnostic centers in each country, with variable participation, which could put the representability of the results in question. Nevertheless, the universal decrease in the number of non-invasive cardiac procedures carried out throughout LATAM suggests that our sample is representative. Finally, no long-term survey data to follow the entire pandemic curve was available at this point in time.

## Conclusion

COVID-19 was associated with a significant and abrupt reduction in cardiac diagnostic procedures in LATAM, which was more related to social distancing measures than to the increase in disease incidence. Better balance and timing of social distancing measures and planning to maintain access to medical care in general and cardiovascular care in particular is warranted during a pandemic surge, especially in regions with high cardiovascular mortality.

## *Supplemental Materials

For additional information, please click here.
